# Diagnostic and Therapeutic Perspectives on Parotid Mammary Analog Secretory Carcinoma: Two Case Reports and Literature Review

**DOI:** 10.1155/crot/5512895

**Published:** 2026-09-26

**Authors:** Francesco Ferragina, Ida Barca, Giuseppe Tarallo, Angelo R. Sottile, Maria Grazia Ioppolo, Maria Giulia Cristofaro

**Affiliations:** ^1^ Department of Experimental and Clinical Medicine, Magna Graecia University, Catanzaro, Italy, unicz.it; ^2^ Department of Neurosciences, Reproductive and Odontostomatological Sciences, Federico II University, Naples, Italy, unina.it

**Keywords:** case report, immunohistochemistry, mammary analog secretory carcinoma, parotid gland tumors, salivary gland tumors

## Abstract

Mammary analog secretory carcinoma (MASC) is a rare malignant tumor of the salivary glands that exhibits significant morphological, immunohistochemical, and molecular similarities with secretory carcinoma of the breast, including the characteristic *ETV6*–*NTRK3* gene fusion. The diagnosis of this condition remains challenging due to its histopathologic overlap with other low‐grade salivary gland tumors, such as acinic cell carcinoma and mucoepidermoid carcinoma. The following report details two cases of MASC manifesting in the right parotid gland of a 37‐year‐old male and a 23‐year‐old female. Both subjects exhibited slow‐growing, painless swellings and radiologic findings indicative of benign lesions. The surgical excision procedure was performed on the first patient via extracapsular dissection, while the second patient underwent superficial parotidectomy. Both procedures were carried out under the supervision of intraoperative facial nerve monitoring. A subsequent histopathological and immunohistochemical examination revealed a glandulocystic growth pattern with eosinophilic secretions and strong immunoreactivity for CK7, S100, and mammaglobin. Although molecular testing for the *ETV6*–*NTRK3* fusion was not performed, the clinical, morphological, and immunophenotypic findings supported the diagnosis of low‐grade secretory carcinoma. The patients exhibited uncomplicated recovery, with transient facial nerve paresis in the second patient resolving within 3 months. The first patient remains disease‐free after 40 months of clinical and radiological follow‐up, while the second patient has shown no evidence of recurrence after 21 months of follow‐up. The cases under consideration serve to emphasize the importance of integrating morphology and immunohistochemistry in routine practice, with molecular confirmation remaining the diagnostic gold standard whenever it is available. It is imperative to be aware of this entity to ensure an accurate diagnosis, optimal surgical management, and appropriate long‐term surveillance. Emerging evidence suggests that targeted therapy with NTRK inhibitors may represent a promising option for advanced or recurrent disease.

## 1. Introduction

Mammary analog secretory carcinoma (MASC) is a rare and recently characterized salivary gland tumor first described by Skálová et al. in 2010 [1]. It exhibited morphologic, immunohistochemical, and molecular similarities with secretory carcinoma of the breast. The most notable of these similarities was the presence of the pathognomonic *ETV6*–*NTRK3* gene fusion, which resulted from the *t*(12;15)(p13;q25) translocation. This genetic hallmark leads to constitutive activation of tyrosine kinase signaling and defines MASC as a distinct low‐grade neoplasm within the spectrum of salivary gland carcinomas [2, 3].

Salivary gland tumors account for approximately 3%–4% of all head and neck neoplasms, and about 80% of them are benign, most commonly pleomorphic adenoma and Warthin’s tumor [4]. Malignant tumors of the parotid gland are uncommon and often present as slow‐growing, painless, and well‐circumscribed masses that can mimic benign lesions. The presence of symptoms such as pain, ulceration, or facial nerve dysfunction can serve as indicators of a heightened probability of malignancy. However, the clinical and radiologic features of MASC are nonspecific, leading to frequent misdiagnosis as acinic cell carcinoma (AciCC), mucoepidermoid carcinoma, or other low‐grade entities [5, 6].

Fine‐needle aspiration cytology (FNAC) and imaging modalities such as ultrasonography and magnetic resonance imaging (MRI) may facilitate the characterization of lesion morphology and its relationship with adjacent structures; however, these methods are inadequate for establishing a definitive diagnosis. Histopathological and molecular evaluation remains essential components of this assessment. Typically, MASC manifests as a microcystic or papillary‐cystic growth pattern, characterized by an abundant eosinophilic cytoplasm and colloid‐like secretions. Immunohistochemistry reveals strong positivity for S100, CK7, and mammaglobin, while the detection of the *ETV6*–*NTRK3* gene fusion by fluorescence in situ hybridization (FISH) or reverse transcription polymerase chain reaction (RT‐PCR) serves to confirm the diagnosis [1, 7].

Recent studies have fortified the molecular and clinicopathologic characterization of this tumor. Han et al. reported that all 13 patients in their cohort tested positive for *ETV6*–*NTRK3*, thereby supporting its role as a reliable diagnostic biomarker [8]. A 2024 single‐center experience from Verona, Italy, demonstrated that several salivary gland carcinomas initially classified as AciCC showed *NTRK3* rearrangements upon molecular testing [9]. These findings underscore the significance of incorporating routine genetic analysis to avert misclassification and to identify patients who may benefit from targeted therapy. Furthermore, updated reviews of salivary gland neoplasms emphasize how molecular profiling is reshaping the current WHO classification, recognizing MASC as a distinct and clinically relevant entity [10].

Although MASC generally manifests as an indolent course and is associated with a favorable prognosis following complete surgical excision, cases of local recurrence, nodal metastasis, and high‐grade transformation have been documented [11–13]. Given the rarity and diagnostic complexity of this tumor, awareness of it is essential for otolaryngologists, pathologists, and head and neck surgeons.

In this report, we present two cases of MASC of the parotid gland diagnosed at our Maxillofacial Surgery Unit in Southern Italy. Furthermore, a narrative review of the literature is provided to summarize the current evidence regarding the clinical presentation, diagnostic challenges, histopathological and molecular characteristics, surgical management, prognosis, and emerging targeted therapies for this uncommon neoplasm. The objective of the literature review was to establish a clinical context for the cases presented, rather than to provide a systematic review of the available evidence.

## 2. Cases Presentation

### 2.1. Case N.1

In December 2022, a 37‐year‐old Caucasian male presented to the Maxillofacial Unit of University “Magna Graecia” of Catanzaro. The patient reported a swelling in the right pretragic region for 1 year, which had gradually increased in volume over the months. The patient’s medical history did not reveal any notable conditions, nor was he undergoing any pharmacological treatment. Physical examination revealed a 2 × 2.5 cm neoformation with tensile elasticity, a smooth surface, regular margins, and no pain. It exhibited adherence to deeper planes and mobility relative to superficial planes. Furthermore, it was covered by healthy skin. No deficits in the mimetic muscles were observed bilaterally.

**FIGURE 1:**
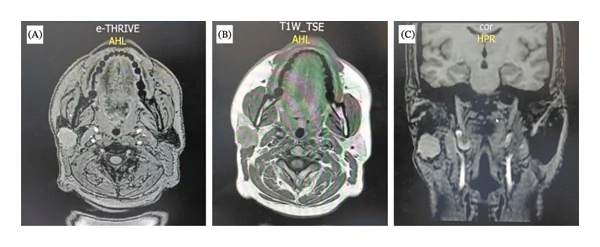
(A) e‐THRIVE, (B) T1W‐TSE axial scans, and (C) coronal scan showing a well‐defined, multilobulated mass in the posteroinferior pole of the right parotid gland.

He underwent a head and neck MRI, which revealed a nodular formation in the posteroinferior pole of the right parotid gland, without evidence of infiltration. The dimensions of the lesion were 3 × 2.5 × 2.7 cm. Its margins were well defined, and its multilobulated structure was complex. The lesion exhibited heterogeneous characteristics, manifesting as hypointense regions on both T1‐ and T2‐weighted images, in conjunction with hyperintense areas on T2‐weighted images, indicative of liquid content. The lesion exhibited significant uptake of the contrast medium, and diffusion‐weighted imaging (DWI) sequences revealed areas of diffusion restriction. The MRI also exhibited small lymph nodes, measuring up to 1 cm, bilaterally in the lateral cervical regions, the largest of which was located in the right angle–mandibular area (Figure 1). These findings suggested the presence of pleomorphic adenomas in the right parotid region. The patient was advised to undergo an FNAC for further diagnostic evaluation; however, he declined the procedure.

**FIGURE 2:**
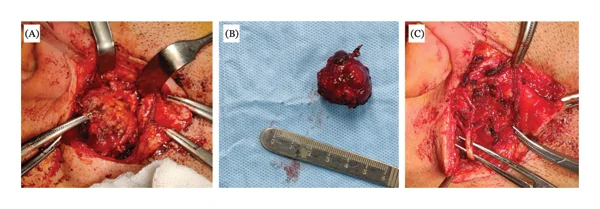
(A, B) Intraoperative macroscopic view; the specimen collected was darker in color than the surrounding gland, and its cut surface demonstrated a firm consistency. (C) Intraoperative macroscopic view of the intact facial nerve after removal of the lesion.

In February 2023, the patient underwent a tumor removal procedure under general anesthesia. An extracapsular dissection was performed with intraoperative facial nerve monitoring to ensure its preservation. The neoformation was excised en bloc, intact, with macroscopically clear margins, and was sent to the Department of Pathological Anatomy for histological examination (Figure 2).

The postoperative course proceeded smoothly; the patient exhibited minor edema of the treated region, with no evidence of macroscopic infection, fluid collections, or facial nerve injury.

A pathological examination confirmed a maximum tumor dimension of 2.8 cm. Surgical margins were clear (R0), with the closest microscopic margin measuring 1.8 mm. There was no evidence of perineural invasion (PNI) or lymphovascular invasion (LVI). The pathological staging was determined as pT2 pNx cM0 (Stage II, according to the Eighth Edition of the AJCC Cancer Staging Manual).

**FIGURE 3:**
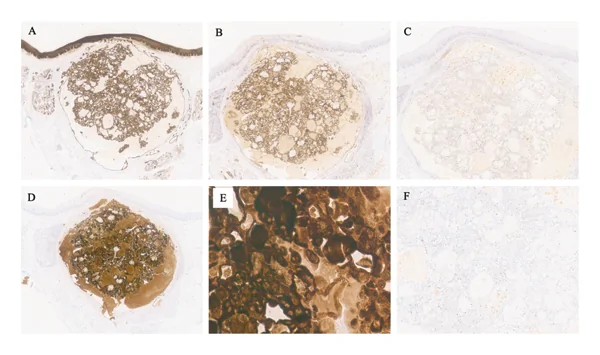
Histopathological features of secretory carcinoma (MASC) of the parotid gland (A) HMW‐CK (high‐molecular‐weight cytokeratin), ×1. Low‐power view shows a neoplastic nodule with diffuse cytoplasmic positivity, which delineates the microcystic/glandulocystic growth pattern; the nodule is characterized by variably sized cystic spaces containing eosinophilic secretory material. (B) S100 protein, ×1. Low‐power view shows diffuse, strong nuclear and cytoplasmic positivity throughout the tumor nodule, consistent with the immunophenotype of secretory carcinoma. (C) GATA3, ×2. Low‐power view shows focal and weak nuclear positivity in a minor subset of cells. (D) Mammaglobin, ×1. Low‐power view demonstrates that the tumor nodule exhibits strong and diffuse cytoplasmic positivity, clearly delineated from the surrounding non‐neoplastic tissue. (E) Mammaglobin, ×40. High‐power view reveals the presence of strong and diffuse granular cytoplasmic positivity in the tumor cells that line the microcystic spaces. (F) Ki‐67, ×4. The proliferation index is low (< 5% in the selected hotspot), with scattered positive nuclei observed predominantly within areas of solid growth.

The histological analysis revealed a tumor exhibiting both solid and glandular‐cystic growth patterns. The tissue under investigation was found to consist of large cellular elements with ample eosinophilic cytoplasm and a dense fibrovascular stroma, with areas of lymphoid infiltrate and peripheral infiltration of fibroadipose stroma. There is an absence of mitotic activity or perineural infiltration. Immunohistochemical studies revealed the following: diffuse positivity for pancytokeratin AE1/AE3 and high‐molecular‐weight keratin (Figure 3A); strong, diffuse positivity for S100 (Figure 3B) and mammaglobin (Figure 3D,E); focal, weak positivity for GATA3 (Figure 3C); and a low Ki‐67 proliferation index (Figure 3F), together with CK7 and CK8/18 positivity and thyroglobulin negativity.

The diagnosis of low‐grade secretory carcinoma (MASC of salivary glands) was made based on histopathological morphology and a classical immunohistochemical profile. The histopathological examination confirmed tumor‐free (negative) surgical margins. Molecular confirmation of the *ETV6*–*NTRK3* gene fusion was not performed, representing a recognized limitation.

Since preoperative MRI features were similar to those of a benign pleomorphic adenoma, and the patient refused a preoperative FNAC, an extracapsular dissection under continuous facial nerve monitoring was initially performed. After a thorough review, the Multidisciplinary Head and Neck Tumor Board determined that the patient’s low‐grade secretory carcinoma had completely clear margins (R0). Given the low‐grade histology, complete resection, and absence of adverse pathologic features (such as LVI or PNI), completion parotidectomy and adjuvant therapy were deemed unnecessary. Instead, a strict clinical and radiological surveillance protocol was established.

To date, there are no specific guidelines available for this malignancy. For this reason, it was decided to follow up on a low‐grade malignant tumor of the salivary glands. A postoperative chest CT scan was performed, which was negative for heteroplasia. Consequently, a head and neck MRI was requested 3 months after surgery, which only demonstrated the scarring outcomes of the performed surgery. For the initial two‐year period, an MRI was conducted every 6 months, with ultrasound examinations of the neck and salivary glands performed between these scans. The patient was also subjected to oncological and radiotherapy evaluation; however, no indications were given for adjuvant treatments. He is still undergoing clinical and instrumental monitoring within our department. At the most recent follow‐up (June 2026), corresponding to 40 months after surgery, the patient was found to be free of local recurrence or distant disease. The subsequent follow‐up procedure entailed a clinical examination, periodic neck ultrasonography, and contrast‐enhanced MRI.

### 2.2. Case N.2

**FIGURE 4:**
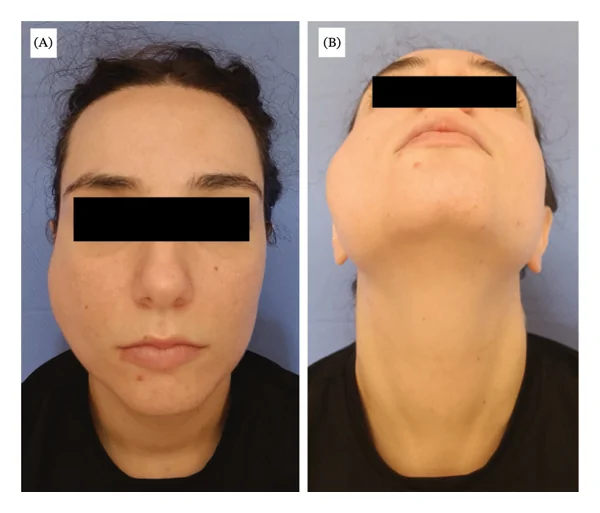
(A, B) Frontal and submental vertex views showing marked swelling in the right parotid region.

In August 2024, a 23‐year‐old Caucasian female presented to the Maxillofacial Unit of University “Magna Graecia” of Catanzaro. The patient reported a swelling in the right pretragic region for almost 1 year, which had gradually increased in volume over the months. The patient’s medical history revealed no record of pathologies or treatment with medications. Physical examination revealed a 3.5‐cm neoformation with a smooth surface, regular margins, and adherence to both deeper and superficial planes (Figure 4). No pain was observed, and no deficits were identified in the mimetic muscles on either side of the body.

The patient was advised to undergo a FNAC test for further diagnostic evaluation; however, she declined the procedure.

**FIGURE 5:**
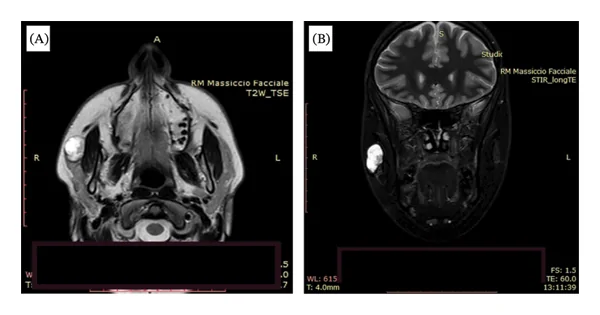
(A) axial and (B) coronal scans showing a well‐defined, multilobulated mass in the upper portion of the right parotid gland.

She underwent a head and neck MRI, which revealed the presence of a nodular formation in the upper portion of the right parotid gland, without evidence of infiltration. The lesion measured 4 × 3 × 2 cm, and its margins were well defined. The MRI scan revealed heterogeneous signal characteristics, with hypointense regions on both T1‐ and T2‐weighted sequences, interspersed with hyperintense areas on T2‐weighted images, consistent with liquid components. Following the administration of contrast medium, the lesion exhibited significant enhancement on imaging, while DWI revealed focal areas of diffusion restriction (Figure 5). The primary diagnostic hypothesis was the presence of a Warthin tumor. The patient was advised to undergo an FNAC for further diagnostic evaluation; however, she declined the procedure.

In October 2024, the patient underwent a superficial parotidectomy procedure under general anesthesia. An intraoperative monitoring system was used to check the integrity of the facial nerve during surgery. The neoformation was excised en bloc, intact, with macroscopically clear margins, and was sent to the Department of Pathological Anatomy for histological examination.

The postoperative course proceeded smoothly; the patient exhibited minor edema of the treated region, with no evidence of macroscopic infection or fluid collections. In the immediate postoperative period, the patient exhibited a mild, transient weakness of the marginal mandibular branch of the facial nerve (House–Brackmann Grade II), ipsilateral to the lesion. The patient was treated with a short course of oral corticosteroids (betamethasone 4 mg, administered twice daily for an average of 7 days), a neurotrophic dietary supplement containing alpha‐lipoic acid 600 mg and B‐complex vitamins (1 tablet daily for 20 days per month for 2 cycles), and physiotherapy. Complete recovery of facial nerve function was documented at 3 months postsurgery. The recovery of facial nerves in these cases is influenced by multiple factors and is indicative of effective management of transient neuropraxia, rather than direct drug efficacy.

A pathological examination confirmed a maximum tumor dimension of 3.2 cm. Surgical margins were determined to be microscopically negative (R0), with the closest clear margin measuring 2.1 mm. PNI and LVI were absent. The pathological staging was classified as pT2 pNx cM0 (Stage II, according to the Eighth Edition of the AJCC Cancer Staging Manual).

**FIGURE 6:**
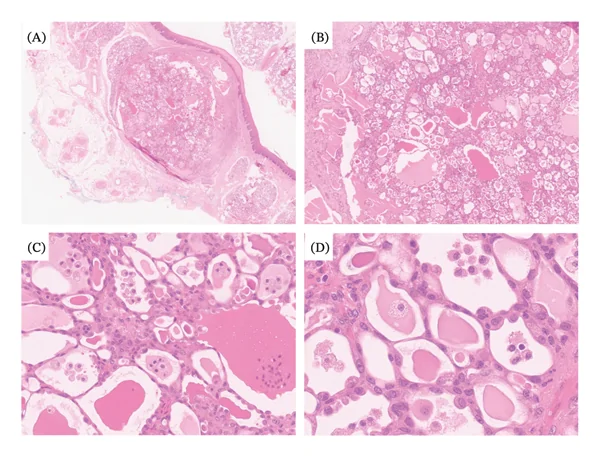
Histopathological features of secretory carcinoma (MASC) of the parotid gland. (A) H&E, ×2. Scanning‐power view showing multiple well‐circumscribed tumor nodules with a predominantly microcystic and glandulocystic architecture, sharply demarcated from the adjacent salivary gland parenchyma. (B) H&E, ×4. Low‐power view showing the microcystic and papillary‐cystic growth pattern, with variably dilated glandular spaces containing eosinophilic secretory material and separated by delicate fibrovascular septa. (C) H&E, ×20. High‐power view showing a cystically dilated space containing dense eosinophilic secretions and lined by tumor cells with abundant eosinophilic‐to‐vacuolated cytoplasm and bland, round nuclei. (D) H&E, ×40. High‐power view demonstrating tumor cells with abundant eosinophilic‐to‐vacuolated cytoplasm and relatively uniform nuclei with inconspicuous nucleoli, arranged around microcystic spaces containing eosinophilic secretory material. No significant nuclear atypia or mitotic figures are identified.

Histopathological examination revealed a predominantly microcystic and glandulocystic neoplasm, composed of relatively uniform cells with round nuclei, slight nucleoli, and abundant eosinophilic to vacuolated cytoplasm. The cystic and glandular spaces contained dense eosinophilic, colloid‐like secretory material and were separated by delicate fibrovascular septa. No significant nuclear atypia or mitotic activity was identified. Representative histopathological findings are demonstrated in Figure 6. Immunohistochemical analysis demonstrated positivity for S100, CK19, and mammaglobin, while GATA3, p63, TTF1, and HBME1 were negative.

The diagnosis of low‐grade secretory carcinoma (MASC of salivary glands) was made based on histopathological features and a classical immunohistochemical profile. Microscopic examination confirmed R0 surgical margins. Molecular confirmation of the *ETV6*–*NTRK3* gene fusion was not performed, representing a recognized limitation.

The patient is still undergoing clinical and instrumental monitoring. The postoperative chest CT scan and the postoperative head and neck MRI both yielded negative results for heteroplasia or disease recurrence. The patient was also subjected to oncological and radiotherapy evaluation; however, no indications were given for adjuvant treatments. She is still undergoing clinical and instrumental monitoring within our department. At the most recent follow‐up (July 2026), corresponding to 21 months after surgery, the patient was found to be free of local recurrence or distant disease. The subsequent follow‐up procedure entailed a clinical examination, periodic neck ultrasonography, and contrast‐enhanced MRI.

## 3. Discussion

MASC is a relatively recent entity among salivary gland malignancies, defined by its characteristic *ETV6*–*NTRK3* gene fusion and histologic overlap with other low‐grade tumors, such as AciCC. For years, many of these tumors were misclassified until Skálová et al. first characterized MASC as a distinct tumor in 2010, establishing a molecular link between it and secretory breast carcinoma [1]. Since its initial recognition, MASC has been increasingly reported in major and minor salivary glands, most frequently in the parotid gland, but also in less common locations such as the submandibular, labial, and palatal glands [6, 14–16]. Despite an increasing number of case reports and small case series, the true incidence remains uncertain, and diagnostic challenges persist due to the tumor’s close morphological resemblance to AciCC and other low‐grade entities [15, 17]. Moreover, a systematic review by Khalele (2017) emphasized that numerous cases previously diagnosed as AciCC were later reclassified as MASC following molecular confirmation, underlining the importance of genetic testing in diagnostic accuracy [18].

### 3.1. Epidemiology and Clinical Presentation

Typically, MASC manifests in adults between the fourth and sixth decades of life, exhibiting no clear gender predominance. However, cases in pediatric and adolescent populations have also been documented. Its estimated incidence is approximately 4% [5, 6, 19]. Clinically, patients typically present with a painless, slowly growing mass in the parotid or other salivary glands, often mimicking benign neoplasms such as pleomorphic adenoma or Warthin’s tumor [14, 20]. Imaging studies, including ultrasound and MRI, generally show well‐circumscribed, lobulated lesions with mixed solid and cystic components. However, these findings are nonspecific and primarily useful for surgical planning rather than diagnosis [21]. In both cases, initial imaging suggested a benign etiology, emphasizing the diagnostic uncertainty that often surrounds this entity.

### 3.2. Histopathological and Molecular Features

Histologically, MASC manifests a solid, microcystic, or papillary‐cystic architecture, with tumor cells containing abundant eosinophilic or vacuolated cytoplasm and colloid‐like secretions [1, 17]. The immunohistochemical profile is distinctive, showing positivity for S100 protein, mammaglobin, vimentin, CK7, and often GATA3, while markers such as DOG1, p63, and SOX10 are typically negative, helping to distinguish it from AciCC and mucoepidermoid carcinoma [19, 22]. Moreover, in a detailed study, Skálová et al. reported three cases of high‐grade transformation within MASC, each resulting in increased expression of both EGFR and β‐catenin, thus providing a valuable indication of the potential molecular pathways involved in the progression of the disease [23].

The definitive diagnostic hallmark remains the detection of the *ETV6*–*NTRK3* gene fusion, identifiable by FISH or RT‐PCR [1, 24, 25]. This translocation results in constitutive activation of the tyrosine kinase signaling pathway, promoting tumorigenesis and providing a potential therapeutic target [23]. The necessity for molecular testing has been highlighted by studies such as those by Urano et al. and Majewska et al., who demonstrated that a considerable proportion of tumors previously classified as AciCC were, in fact, MASC upon genetic analysis [6, 26]. Furthermore, Skálová et al. reported cases harboring alternative *ETV6* fusion partners (*ETV6-X*), suggesting molecular heterogeneity that may correlate with more aggressive biological behavior [23]. According to Boom et al., who analyzed 31 patients with MASC, the presence of the *ETV6*–*NTRK3* fusion correlates with a very favorable clinical course, with low chances of local recurrences, very rare metastases, and high survival rates [27].

In the present report, molecular testing was not available. Consequently, the diagnosis in both patients was based on the characteristic morphological features and immunohistochemical profile, which provided substantial support for the diagnosis of secretory carcinoma. It is acknowledged that the absence of molecular confirmation represents a limitation of the present study.

### 3.3. Differential Diagnosis

Given the overlapping cytological and architectural features, a differential diagnosis of MASC from AciCC, mucoepidermoid carcinoma, and low‐grade cribriform cystadenocarcinoma is imperative [15, 17]. The distinction is clinically relevant, as MASC generally exhibits a more indolent course but may show local recurrence or, rarely, high‐grade transformation [23, 28]. The differential diagnosis focused primarily on distinguishing MASC from AciCC and mucoepidermoid carcinoma. AciCC was excluded based on the absence of intracytoplasmic PAS‐D–positive zymogen granules, negative/absent DOG1 expression, and strong diffuse reactivity for S100 and mammaglobin. Mucoepidermoid carcinoma was ruled out by the lack of epidermoid/squamoid differentiation and complete negativity for p63 and p40. Although FNAC is a useful diagnostic tool, it is not sufficiently reliable to distinguish MASC from its mimics, particularly in the absence of molecular testing [19, 20]. Immunohistochemistry for S100 and mammaglobin can raise suspicion, but confirmation through *ETV6*–*NTRK3* detection is recommended whenever feasible [22, 24].

In 2015, Urano et al. reclassified 10 cases of AciCC that had previously been diagnosed as MASC following molecular testing. This study highlighted the diagnostic pitfalls involved and emphasized the importance of routine genetic analysis [26]. Furthermore, Ito et al. described 14 cases of MASCs with *ETV6* rearrangement, two of which involved alternative fusion partners (*ETV6-X*), indicating molecular variability that could have prognostic implications [3]. These results underscore the necessity for FISH or RT‐PCR to substantiate the diagnosis, particularly in cases where histological evaluation is equivocal.

### 3.4. Management and Prognosis

The standard treatment for MASC involves complete surgical excision with histologically negative margins [14, 17]. The extent of surgery, which can range from extracapsular dissection to superficial or total parotidectomy, is contingent upon factors such as tumor size, location, and involvement of adjacent structures [21, 29]. Preserving the functionality of nerve structures is of equal importance, and this objective can be achieved by intraoperative monitoring of said structures [30]. Neck dissection is not generally recommended unless there is clinical or radiological evidence of nodal metastasis [14]. Adjuvant radiotherapy may be considered in cases of high‐grade transformation, positive margins, or regional spread, although data are limited. Chemotherapy is rarely indicated and lacks standardized protocols [28].

In both presented cases, complete surgical excision was performed, accompanied by intraoperative monitoring of the facial nerve to preserve its function. The postoperative course was uneventful in the first case, while the second patient experienced transient facial nerve paresis that resolved with physiotherapy within 3 months. During the subsequent follow‐up period, neither patient exhibited signs of recurrence, which is consistent with the typically indolent clinical course reported for localized, low‐grade secretory carcinomas following complete surgical resection [14, 28].

Although secretory carcinoma is a rare form of malignant neoplasm, its surgical management should be interpreted within the broader context of parotid gland neoplasms. Most parotid tumors are benign and are commonly managed by superficial parotidectomy or extracapsular dissection, depending on tumor characteristics and surgeon experience. However, as demonstrated by the cases presented here, it is important to note that lesions which initially appear to have benign clinical and radiological features may ultimately prove to be malignant on definitive histopathological examination. This highlights the indispensable role of postoperative pathological assessment in establishing the final diagnosis. Moreover, the preservation of facial nerve function remains a primary objective of parotid surgery, irrespective of the histology of the tumor. Although transient facial nerve dysfunction is among the most frequent postoperative complications, careful surgical planning, intraoperative facial nerve monitoring, and appropriate postoperative follow‐up contribute to favorable functional recovery and optimal oncological outcomes. These findings are consistent with the experience reported in large surgical series of parotid gland neoplasms, which emphasize the importance of balancing oncologic radicality with facial nerve preservation while recognizing the crucial role of final histopathological evaluation in guiding postoperative management [31].

### 3.5. Emerging Role of Targeted Therapy

The MASC can serve as a valuable model for comprehending the scope and constraints of precision oncology in the context of rare tumors. The recurrent presence of the *ETV6*–*NTRK3* gene fusion, identified as the primary driver event in the majority of MASC cases, has given rise to the possibility of targeted treatment through selective inhibition of TRK proteins. In fact, recent advancements in molecular oncology have led to the identification of potential targeted treatment options for MASC. The identification of the *ETV6*–*NTRK3* gene fusion designates these tumors as candidates for therapy with TRK inhibitors such as larotrectinib and entrectinib, which have demonstrated promising efficacy in tumors harboring *NTRK* gene fusions across multiple histologic types [32–34].

Clinical trials and case reports have demonstrated durable responses to TRK inhibitors such as larotrectinib and entrectinib in patients with NTRK fusion‐positive solid tumors, including secretory carcinoma. However, potential acquired resistance mechanisms (such as *NTRK3* solvent‐front mutations) remain a clinical challenge [32]. These findings underscore the therapeutic potential and the challenges posed by resistance mechanisms, suggesting that molecularly guided management may become increasingly pertinent for patients with unresectable, recurrent, or metastatic MASC [33, 35, 36].

In a broader context, Cardona et al. and Ignatova et al. have highlighted the significance of targeted therapies directed against *NTRK1/2/3* fusions, emphasizing their role as a paradigm of a tumor‐agnostic approach, in which the therapeutic decisions are guided by molecular alterations rather than by the histological origin of the tumor. This model has proven to be particularly relevant for rare tumors such as MASC, in which the molecular definition can have a disproportionate clinical impact on their incidence.

### 3.6. Prognostic Considerations and Follow‐Up

Although MASC is generally associated with a favorable outcome, cases of high‐grade transformation and distant metastasis have been documented [23, 28, 37, 38]. Documentation of aggressive variants with poor prognosis by Skálová et al. and Luo et al. underscores the importance of long‐term surveillance, even in low‐grade tumors [23, 38]. The following risk factors for recurrence have been identified: positive surgical margins, PNI, and the presence of *ETV6-X* fusion variants [23]. Consequently, it is recommended that close clinical and imaging follow‐up be conducted, particularly during the initial 2 years following surgery [14, 28].

Although secretory carcinoma generally follows a relatively slow and stable clinical course, rare cases involving positive surgical margins, regional lymph node metastases, distant dissemination, or high‐grade transformation have been documented. These aggressive presentations underscore the significance of individualized surgical planning, multidisciplinary discussion, and the consideration of adjuvant treatment in selected patients. Consequently, long‐term clinical and radiological follow‐up remains essential even after apparent complete surgical excision, as emphasized by recent reports of metastatic secretory carcinoma [39].

## 4. Conclusions

Secretory carcinoma (formerly MASC) is a rare salivary gland malignancy that remains challenging to diagnose because of its considerable clinical, radiological, and histopathological overlap with other low‐grade salivary gland tumors. As demonstrated by the two cases presented here, patients typically present with slowly enlarging, painless parotid masses whose imaging characteristics may suggest a benign lesion, thereby making preoperative diagnosis difficult.

The diagnosis of MASC should ideally rely on an integrated approach combining histopathological evaluation, immunohistochemistry, and molecular analysis for the detection of the *ETV6–NTRK3* fusion. This combination of methods remains the diagnostic gold standard whenever it is available. In the present cases, molecular testing was not performed; therefore, the diagnosis was supported by the characteristic morphological features together with a highly consistent immunohistochemical profile. Despite the ample support for this approach within the extant literature, the absence of molecular confirmation must be recognized as a significant limitation of the present report.

The cornerstone of treatment for localized disease remains complete surgical excision with preservation of facial nerve function. This is generally associated with an excellent prognosis when negative surgical margins are achieved. Nevertheless, it is recommended that careful long‐term clinical and radiological follow‐up is undertaken, given the rare possibility of local recurrence, nodal metastasis, or high‐grade transformation.

Finally, the increased knowledge of the molecular landscape of secretory carcinoma has important therapeutic implications. The identification of *NTRK* gene fusions provides diagnostic confirmation and also identifies patients who may benefit from TRK inhibitor therapy in advanced, recurrent, or unresectable disease. As access to molecular diagnostics becomes more widespread, the integration of histopathology, immunohistochemistry, and genomic testing will further improve diagnostic accuracy and support a more personalized approach to the management of this uncommon salivary gland neoplasm.

## Author Contributions

Conceptualization, Francesco Ferragina and Maria Giulia Cristofaro; methodology, Francesco Ferragina and Ida Barca; investigation and clinical management, Francesco Ferragina, Ida Barca, Giuseppe Tarallo, Angelo R. Sottile, and Maria Grazia Ioppolo; data curation, Francesco Ferragina, Giuseppe Tarallo, and Angelo R. Sottile; writing–original draft preparation, Francesco Ferragina and Giuseppe Tarallo; writing–review and editing, Francesco Ferragina, Ida Barca, and Maria Giulia Cristofaro; visualization, Maria Giulia Cristofaro; supervision, Maria Giulia Cristofaro and Francesco Ferragina.

## Funding

This research received no external funding. Open‐access publishing was facilitated by Universita degli Studi Magna Graecia di Catanzaro, as part of the Wiley–CRUI‐CARE agreement.

## Disclosure

All authors have read and agreed to the published version of the manuscript.

## Ethics Statement

This study was conducted in accordance with the ethical standards outlined in the Declaration of Helsinki on Medical Protocol and Ethics. The study was approved by the Ethics Committee of Magna Graecia University of Catanzaro (protocol no. 280, dated September 17, 2020), which covers ongoing observational data collection for salivary gland pathology encompassing both reported cases (2022 and 2024).

## Consent

Written informed consent for publication of their clinical details and identifiable/deidentified clinical and radiological images was obtained from the subjects involved in the study.

## Conflicts of Interest

The authors declare no conflicts of interest.

## Data Availability

The data that support the findings of this study are available on request from the corresponding author. The data are not publicly available due to privacy or ethical restrictions.
